# An Optimized Triple Modality Reporter for Quantitative *In Vivo* Tumor Imaging and Therapy Evaluation

**DOI:** 10.1371/journal.pone.0097415

**Published:** 2014-05-09

**Authors:** Rachel A. Levin, Csilla N. Felsen, Jin Yang, John Y. Lin, Michael A. Whitney, Quyen T. Nguyen, Roger Y. Tsien

**Affiliations:** 1 Department of Pharmacology, UCSD School of Medicine, University of California San Diego, La Jolla, California, United States of America; 2 Division of Otolaryngology/Head and Neck Surgery, University of California San Diego, La Jolla, California, United States of America; 3 Howard Hughes Medical Institute, La Jolla, California, United States of America; Hormel Institute, University of Minnesota, United States of America

## Abstract

We present an optimized triple modality reporter construct combining a far-red fluorescent protein (E2-Crimson), enhanced firefly luciferase enzyme (Luc2), and truncated wild type herpes simplex virus I thymidine kinase (wttk) that allows for sensitive, long-term tracking of tumor growth *in vivo* by fluorescence, bioluminescence, and positron emission tomography. Two human cancer cell lines (MDA-MB-231 breast cancer and HT-1080 fibrosarcoma cancer) were successfully transduced to express this triple modality reporter. Fluorescence and bioluminescence imaging of the triple modality reporter were used to accurately quantify the therapeutic responses of MDA-MB-231 tumors to the chemotherapeutic agent monomethyl auristatin E *in vivo* in athymic nude mice. Positive correlation was observed between the fluorescence and bioluminescence signals, and these signals were also positively correlated with the *ex vivo* tumor weights. This is the first reported use of both fluorescence and bioluminescence signals from a multi-modality reporter construct to measure drug efficacy *in vivo*.

## Introduction

The development of noninvasive imaging technologies has significantly advanced the field of molecular imaging. Molecular imaging is commonly used to analyze cancer initiation and cancer growth pathways as well as therapeutic responses in living subjects [1]. However, each molecular imaging modality has strengths and weaknesses. We combined a fluorescent protein gene for fluorescence imaging, a luciferase gene for bioluminescence imaging, and a thymidine kinase gene for positron emission tomography (PET) scanning into a triple modality reporter construct, wherein the three components offer complementary advantages that compensate for the disadvantages of each individual component.

The first component of our optimized triple reporter construct is the far-red fluorescent protein E2-Crimson (excitation maximum 611 nm, emission maximum 646 nm) for fluorescence imaging. Fluorescent proteins can be detected at single cell resolution without requiring the administration of an exogenous substrate. As a result, fluorescent proteins are useful for fluorescent-activated cell sorting (FACS), determining transduction efficiency by microscopy, tumor imaging during fluorescence-guided surgery, and tumor identification and quantification in tissue sections. Previous triple reporter designs have contained the shorter wavelength EGFP (enhanced green fluorescent protein; excitation maximum 488 nm, emission maximum 507 nm) or mRFP1 (monomeric red fluorescent protein; excitation maximum 584 nm, emission maximum 607 nm) [2]–[5]. However, short wavelength fluorescence signal has greater attenuation in mammalian tissue due to the spectral overlap with heme absorbance *in vivo*. Also, mRFP1 was an early monomeric red fluorescent protein, which has been rendered completely obsolete. For this reason, we chose an up-to-date far-red to infrared fluorescent protein to allow for increased penetration of the fluorescence signal in whole animal live imaging. However, several varieties of such proteins existed, so we conducted an unbiased comparison of the cellular expression and *in vivo* signals of E2-Crimson, infrared fluorescent protein (IFP1.4), mNeptune, and mPlum.

The second gene in this triple reporter is enhanced firefly luciferase (Luc2). Luc2 is a codon-optimized luciferase gene that was engineered to have increased expression in mammalian cells. Luc2 reportedly can detect single cancer cells *in vivo* by bioluminescence [6], which is a higher sensitivity than reported for firefly luciferase, *Renilla* luciferase, and mutant thermostable firefly luciferase, all of which have been used in other triple reporter constructs [2]–[5]. Unlike fluorescent proteins, Luc2 requires the external substrate D-luciferin and adenosine triphosphate (ATP) from living cells for the reaction that generates photons of light visible as bioluminescence. Bioluminescence imaging has superior signal-to-background ratios compared to fluorescence imaging due to the absence of significant autoluminescence in mammalian tissues [7]. This superior signal-to-background ratio with bioluminescence allowed for greater sensitivity of imaging, as assessed by a lower number of cells required for *in vivo* tumor detection with bioluminescence compared to fluorescence in the triple reporter.

The third reporter gene in this triple reporter construct is truncated wild type herpes simplex virus I thymidine kinase (wttk), which is used for PET imaging. PET imaging, which is the only method that is routinely used clinically, produces signal with the best depth penetration compared to fluorescence and bioluminescence, and permits accurate three-dimensional reconstruction. However, PET has low cellular resolution and sensitivity; thus, PET quantification is less reliable in small tumors in mice. Additionally, wttk requires the administration of the radiolabelled probe 9-(4-^18^F-fluoro-3-[hydroxymethyl]butyl)guanine, referred to as ^18^F-FHBG, to produce signal. Wttk phosphorylates ^18^F-FHBG by transferring a phosphate group from ATP, which traps ^18^F-FHBG in cells expressing wttk, allowing for PET imaging of wttk-expressing tumors [8].

Previous triple reporter constructs have consisted of large fusion proteins or contained internal ribosomal entry sites (IRES) [2]–[5] to facilitate the co-expression of the three reporter genes. Large fusions of all three reporter proteins can affect protein folding and/or trafficking as well as reduce protein activity [5], [9]. Also, IRES-dependent gene expression is often significantly less efficient than the 5′ cap-dependent promoter-driven expression of the preceding gene [10]. Instead, we used ‘self-cleaving’ viral 2A sequences from porcine teschovirus-1 (P2A) and *Thosea asigna* virus (T2A), where translational disruption between the glycine and proline residues in the D-X-E-X-N-P-G↓P consensus sequence yields expression of separate proteins from a single multicistronic mRNA. The 2A sequence-mediated ribosomal ‘skip’ allows for efficient expression of multiple genes irrespective of their order in the construct [11]. All three proteins should be transcribed at equimolar amounts, though their steady-state levels will vary according to their individual degradation rates. A Gly-Ser-Gly linker precedes both 2A sequences for optimal cleavage [12].

Our optimized triple reporter was expressed and validated *in vitro* and *in vivo*, in HEK293A human embryonic kidney, MDA-MB-231 human breast cancer, and HT-1080 human fibrosarcoma cell lines. Finally, we were able to accurately assess the therapeutic responses of the MDA-MB-231 human breast cancer cell line expressing the triple reporter *in vivo* to the chemotherapeutic agents monomethyl auristatin E and F (MMAE and MMAF) with fluorescence and bioluminescence imaging.

## Results

**Figure 1:**
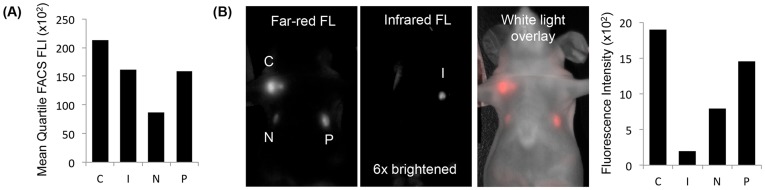
Comparison of four far-red and infrared fluorescent proteins. (A) The mean quartile (mean 25%) fluorescence intensities (FLI) of E2-Crimson (C), infrared fluorescent protein (I), mNeptune (N), and mPlum (P) in HT-1080 cells measured by fluorescent-activated cell sorting (FACS) were compared (100 mW laser with ex 568 nm and em 650–670 nm for C, N, and P; ex 690 nm and em 710–900 nm for I). (B) The top 5% brightest HT-1080 cells from each fluorescent protein cell type were injected into athymic nude mice (1×10^6^ cells/injection; exposure time: 500 msec; ex 590/23 nm and em 645LP for C, N, and P; ex 640/48 nm and em 700LP for I).

Fluorescent proteins used in previously published triple reporters have been excited below 600 nm, overlapping with heme absorbance, which contributes to depth attenuation. Because E2-Crimson [13] (excitation maximum 611 nm), infrared fluorescent protein [14] (IFP, excitation maximum 684 nm), mNeptune [15] (excitation maximum 600 nm), and mPlum [16] (excitation maximum 590 nm) have all been published separately, the expression of these far-red to infrared FPs in stably transfected cells has not been compared head-to-head *in vivo*. Stable, consistent, and high expression of these proteins is necessary for *in vivo* tumor imaging where fluorescent signal is monitored for weeks to months of tumors growth. Comparing human fibrosarcoma HT-1080 cells stably expressing E2-Crimson, IFP, mNeptune, or mPlum, HT-1080 E2-Crimson cells had the highest mean quartile (mean 25% of cells) fluorescence according to fluorescent-activated cell sorting (FACS) analysis, even though the far-red settings were optimized for mPlum (ex 568 nm and em 650-670 nm; IFP was assessed at ex 690 nm and em 710–900 nm) (Figure 1A). The same was true for HT-1080 tumors *in vivo*, in which HT-1080 E2-Crimson tumors had the highest *in vivo* fluorescence at the far-red imaging settings (ex 590/23 nm and em 645LP for E2-Crimson, mNeptune, and mPlum; ex 640/48 nm and em 700LP for IFP) (Figure 1B). E2-Crimson may be brighter *in vitro* and *in vivo* from the combination of its high quantum yield, tetrameric structure, and rapid folding stability. As a result, E2-Crimson was incorporated into our optimized triple modality reporter design.

**Figure 2:**
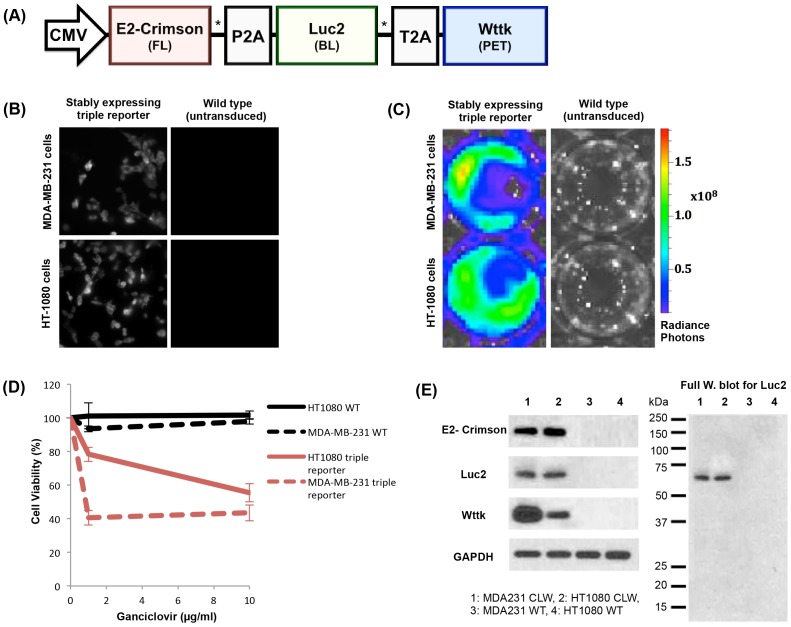
Triple reporter schematic and *in vitro* validation of components. (A) The optimized triple modality reporter construct consists of a far-red fluorescent protein (E2-Crimson) for fluorescence imaging (FL), enhanced firefly luciferase enzyme (Luc2) for bioluminescence imaging (BL), and truncated wild type herpes simplex virus I thymidine kinase (wttk) for positron emission tomography (PET). Viral 2A sequences (P2A and T2A) separate each component. A flexible Gly-Ser-Gly linker (*) precedes each 2A sequence. (B) Epifluorescence microscopy of E2-Crimson (580/20 nm excitation filter, 653/95 nm emission filter, 1 second exposure, 40× oil objective) in the wild-type and triple reporter cell lines (stable expression >3 weeks). Images were scaled equally using ImageJ software. (C) Radiance photons (p/s/cm^2^/sr) produced from Luc2 activity in the triple reporter cell lines (stable expression >3 weeks) compared to their respective wild type cell lines (7.4×10^4^ cells/well) immediately after exposure to D-luciferin (150 µg/ml). The wells shown are representative of triplicate wells in a 48-well plate. (D) Wttk activity measured by cell death after 6 days of ganciclovir treatment for the triple reporter cell lines (stable expression >3 weeks) compared to their respective wild type (WT) cell lines. Only viable cells can convert the CellTiter 96 AQueous One Solution into a product with an absorbance at 490 nm, which was used to calculate the cell viability. (E) Expression of each individual reporter protein in the triple reporter (CLW) cell lines compared to their respective wild type (WT) cell lines shown by Western blot (3×10^4^ cells/well). Successful self-cleavage of viral 2A sequences was observed in all Western blots, as shown in the representative full Western blot for Luc2. A fusion protein consisting of all three modalities (∼135 kDa) or two of the successive modalities (∼95 and ∼106 kDa for E2-Crimson+Luc2 and Luc2+wttk, respectively) was not observed.

The autofluorescence of tissues at excitation wavelengths shorter than heme absorbance has contributed to inferior sensitivity of shorter wavelength fluorescent proteins *in vivo* compared to bioluminescence imaging [7]. Therefore, we hypothesized that the longer wavelength E2-Crimson would have less competition from autofluorescence and may have comparable sensitivity to bioluminescence imaging. We evaluated the *in vivo* detection limits of E2-Crimson and Luc2 in HT-1080 cells stably expressing the triple reporter (Figure 2A) and found that fluorescence imaging could be used to detect as few as 2,500 cells while bioluminescent imaging could be used to detect as few as 500 cells (Figure S1).

For subsequent experiments, lentivirus containing the optimized triple reporter construct (Figure 2A) was used to transduce the MDA-MB-231 human breast cancer and HT-1080 human fibrosarcoma cell lines. Three weeks after transduction, populations of stable cells from the cell lines were selected by FACS for the brightest 1.5% of cells based on E2-Crimson fluorescence. When imaged under the microscope at single cell level, fluorescence was detected for >95% of cells in the field of view (Figure 2B). Although cells were selected based on fluorescence signal only, Luc2 and wttk were active in the stable cells as assayed with bioluminescence and a ganciclovir toxicity assay, respectively (Figure 2C and D).

We confirmed the complete self-cleavage of the viral 2A sequences by Western blot analysis. While individual reporter proteins were clearly detected in both triple reporter cell lines, we did not detect any fusion protein consisting of all three modalities (∼135 kDa) or two of the successive modalities (∼95 and ∼106 kDa for E2-Crimson+Luc2 and Luc2+wttk, respectively) (Figure 2E).

### *In vivo* validation of the triple reporter imaging modalities

**Figure 3:**
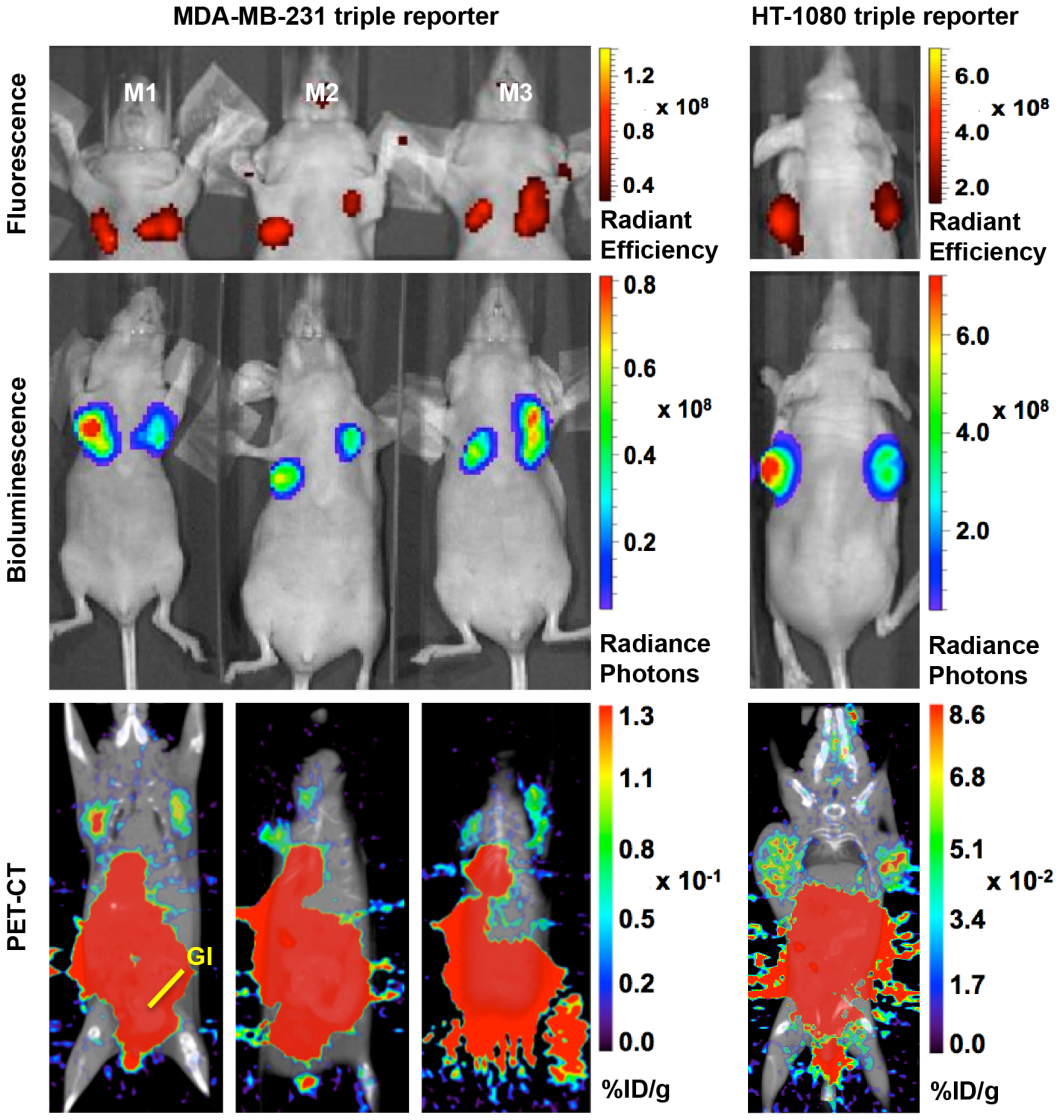
*In vivo* validation of the triple reporter components. Representative live animal images from the coronal plane of six MDA-MB-231 triple reporter tumors and two HT-1080 triple reporter tumors confirm the activity of all three imaging modalities in both cell lines *in vivo.* The fluorescence signal is shown as the radiant efficiency (p/s/cm^2^/str)/(mW/cm^2^). The bioluminescence signal is shown as the radiance photons (p/s/cm^2^/sr). The PET signal is shown as the% injected dose of ^18^F-FHBG per gram. High gut (GI) retention is characteristic of ^18^F-FHBG in microPET imaging, which required that tumors be placed away from the abdomen of each mouse.

Next, the expression of each modality was quantified *in vivo* (Figure 3). Athymic nude mice (female, 6-weeks-old) were injected bilaterally with MDA-MB-231 (1×10^6^ cells/tumor; orthotopic into mammary fat pads) or HT-1080 (5×10^5^ cells/tumor; subcutaneous at shoulder blades) cells that had been previously transduced with the triple reporter. For each cell line imaged, eight triple reporter and four untransduced tumors were generated. There were no significant changes in the growth rate observed between the tumors expressing the triple reporter and their respective untransduced tumors.

All eight MDA-MB-231 triple reporter tumors, ranging from 45.2 to 238.8 mm^3^ based on positron emission tomography–computed tomography (PET-CT) measurements, were healthy and quantifiable by all three modalities. Two HT-1080 triple reporter tumors, which were 446.5 and 520.4 mm^3^ based on PET-CT measurements, were quantifiable by all three modalities (Table 1).

**Table 1:** Sizes and modality signals of the triple reporter tumors after two weeks of growth.

	MDA-MB-231 triple reporter tumor average (n = 8)	HT-1080 triple reporter tumor average (n = 2)
Size measured by caliper (mm^3^)	96.2±42.1	339.0±63.6
Size measured by PET-CT (mm^3^)	118.0±57.4	483.5±52.3
Total fluorescence [(p/s)/(cm^2^/sr)]	3.6×10^8^±1.4×10^8^	1.6×10^9^±5.1×10^8^
Total bioluminescence (p/s)	3.0×10^8^±1.8×10^8^	2.7×10^10^±8.8×10^9^
Total PET (% bioavailable dose)	9.8×10^−2^±6.8×10^−2^	1.7×10^−1^±5.9×10^−2^

The tumor size, fluorescence signal, bioluminescence signal, and PET signal were quantified for the MDA-MB-231 and HT-1080 triple reporter tumors expressing all three modalities *in vivo*. Only tumors that were healthy and large enough to be detectable by all three modalities of the triple reporter construct were averaged and included in this dataset.

One additional HT-1080 triple reporter tumor, measured to be 552.3 mm^3^ by PET-CT, was quantifiable by all three modalities. However, this larger HT-1080 triple reporter tumor was excluded from the data set summarized in Table 1 because it was the only necrotic tumor generated in this study. The dead cells in this tumor were visible by fluorescence, which is likely because stable fluorescent proteins can remain visible in dead cells and debris until they are proteolyzed, but they were not visible by bioluminescence or PET, modalities that require ATP to generate a signal.

The remaining five HT-1080 triple reporter tumors, ranging from ∼18 to 245 mm^3^ based on caliper measurements, were quantifiable by fluorescence and bioluminescence signals but were too small to detect by PET signal, so they were also excluded from the data set summarized in Table 1 (Table S1). Despite their significantly larger size, the average total PET signal (% injected dose) of the HT-1080 triple reporter tumors successfully imaged by all three modalities was not significantly higher than that of the MDA-MB-231 triple reporter tumors (Table 1). The MDA-MB-231 triple reporter cell line was chosen for use in the MMAE and MMAF therapy experiment because this cell line showed more uniform growth *in vivo* than the HT-1080 triple reporter cell line. Furthermore, because the PET measurements of small murine tumors were significantly less sensitive than fluorescence and bioluminescence signals (tumors visible by fluorescence and bioluminescence were not always visible by PET), PET was not used to track the tumor therapeutic responses to MMAE.

### Use of the triple reporter to monitor the response of sub-palpable tumors to therapy

For the therapy experiment, MDA-MB-231 triple reporter cells (7.5×10^5^) were implanted orthotopically into bilateral mammary fat pads of athymic nude mice (female, 5-weeks-old). On day seven post implantation, mice were randomized into three treatment groups (untreated, MMAE-treated, and MMAF-treated, n = 5 mice per group). At this point, tumors were too small to be reliably measured by calipers, but they had reliable fluorescence and bioluminescence signals. We did not measure PET signal, as tumors were smaller than the likely detection limit by PET for tumor imaging in mice. Mice in the MMAE-treated and MMAF-treated groups were treated every three days for a total of six doses of 0.5 nmol/g starting on day seven. We did not expect a therapeutic effect from MMAF treatment, as a charged phenylalanine residue on the C-terminus of MMAF interferes with its intracellular access [17].

**Figure 4:**
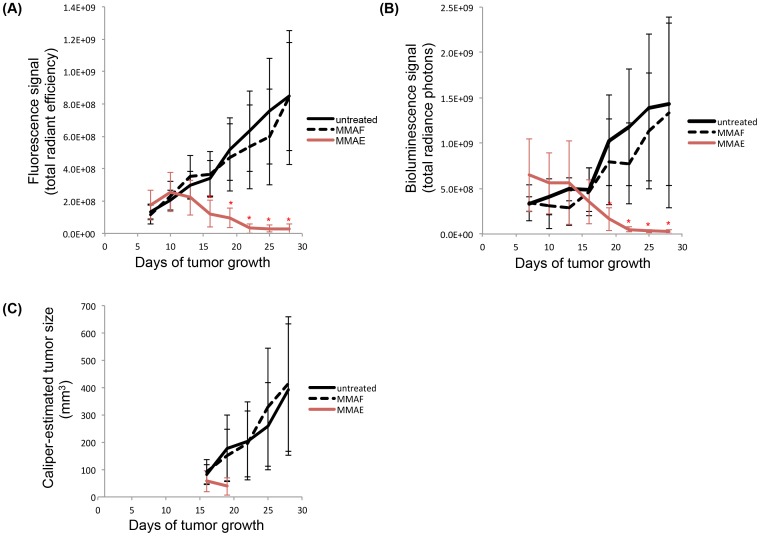
Quantification of the triple reporter optical signals to monitor therapy responses *in vivo*. (A) Average fluorescence signal [(p/s)/(cm^2^/sr)] of each MDA-MB-231 triple reporter tumor treatment group over time. (B) Average bioluminescence signal (p/s) of each MDA-MB-231 triple reporter tumor treatment group over time. (C) Average size (mm^3^) of each MDA-MB-231 triple reporter tumor treatment group over time based on caliper measurements. MMAE or MMAF (0.5 nmol/g) was administered on days 7, 10, 13, 16, 19, and 22. Significant decreases (p<0.005) in the tumor optical signals in the MMAE-treated group compared to the untreated and MMAF-treated groups are indicated by*.

On day 16, a significant decrease in the tumor fluorescence signal (Figure 4A) was detected in the MMAE-treated group (1.2×10^8^±8.2×10^7^ [(p/s)/(cm^2^/sr)]) compared to the untreated (3.4×10^8^±1.1×10^8^ [(p/s)/(cm^2^/sr)]) and MMAF-treated (3.7×10^8^±1.4×10^8^ [(p/s)/(cm^2^/sr)]) groups (p = 5.0×10^−4^). On day 19, a significant decrease in the tumor bioluminescence signal (Figure 4B) was detected in the MMAE-treated group (1.7×10^8^±1.3×10^8^ (p/s)) compared to the untreated (1.0×10^9^±5.0×10^8^ (p/s)) and MMAF-treated (8.0×10^8^±4.7×10^8^ (p/s)) groups (p = 2.0×10^−4^). At no point did the MMAF-treated group show a significant decrease in the tumor fluorescence or bioluminescence signal compared to the untreated group.

On day 16, tumors in the untreated and MMAF-treated groups reached an average size of ∼100 mm^3^. From then on, a caliper was used to track therapy in addition to the fluorescence and bioluminescence signals in the untreated and MMAF-treated groups. Tumors in the MMAE-treated group remained smaller than an average of ∼100 mm^3^ throughout the experiment. Caliper measurements were only attempted for tumors in the MMAE-treated group on days 16 and 19 because of the small sizes of the tumors due to treatment (Figure 4C). For both the untreated and MMAF-treated groups, there was a strong correlation (on days 16–28) between the fluorescence signal and caliper measurements as well as between the bioluminescence signal and caliper measurements (R^2^>0.83 for all cases, data not shown).

**Figure 5:**
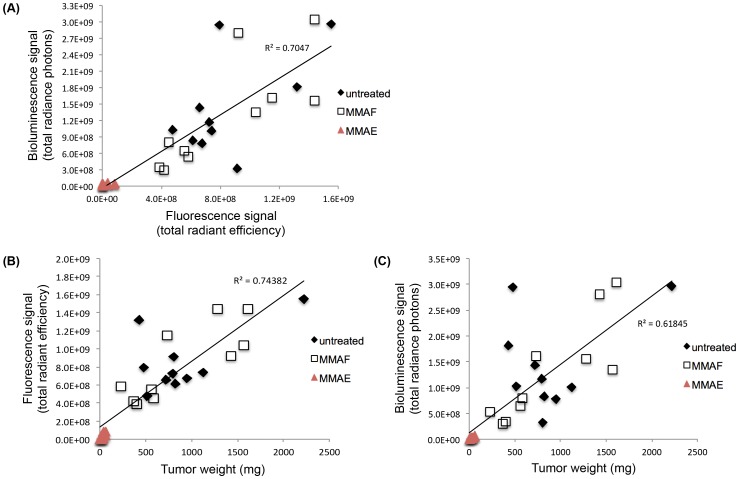
Correlation between triple reporter tumor optical signals *in vivo* and weights *ex vivo* following therapy. (A) Fluorescence signal [(p/s)/(cm^2^/sr)] vs. bioluminescence signal (p/s) of the MDA-MB-231 triple reporter tumors on day 28 of tumor growth. (B) Fluorescence signal [(p/s)/(cm^2^/sr)] vs. weight (mg) of the MDA-MB-231 triple reporter tumors on day 28 of tumor growth. (C) Bioluminescence signal (p/s) vs. weight (mg) of the MDA-MB-231 triple reporter tumors on day 28 of tumor growth.

Four weeks post-implantation, the tumors were harvested and weighed. The final *in vivo* fluorescence and bioluminescence signals of the tumors directly correlated with each other (Figure 5A, R ^2^ = 0.70) as well as with the *ex vivo* weights of the tumors (weight vs. fluorescence signal: Figure 5B, R ^2^ = 0.74; weight vs. bioluminescence signal: Figure 5C, R ^2^ = 0.62; Table 2).

**Table 2:** Final triple reporter tumor optical signals *in vivo* and weights *ex vivo* following therapy.

	Untreated average (n = 10)	MMAF average (n = 10)	MMAE average (n = 10)
Weight (mg)	885.9±416.2	878.3±536.7	29.8±21.4*
Total fluorescence [(p/s)/(cm^2^/sr)]	8.5×10^8^±3.4×10^8^	8.4×10^8^±4.1×10^8^	2.6×10^7^±3.3×10^7^*
Total bioluminescence (p/s)	1.4×10^9^±8.9×10^8^	1.3×10^9^±9.8×10^8^	2.8×10^7^±2.1×10^7^*
Caliper-estimated size (mm^3^)	393.7±237.8	413.4±245.7	Sub-palpable*

Tumors from the therapy experiment were imaged then resected and weighed after 28 days of growth *in vivo*. Optical signals of the triple reporter tumors were representative of the tumor mass. Tumors in the MMAE-treated group were too small to be measured by a caliper after day 19. Significant decreases (p<0.005) in the tumor weight and optical signals in the MMAE-treated group compared to the untreated and MMAF-treated groups are indicated by*.

## Discussion

We report the successful generation of an optimized triple modality reporter (Figure 2A) and viral transduction of this triple reporter construct into two human cancer cell lines, MDA-MB-231 and HT-1080. Both transduced cell lines expressed all three modalities *in vitro* (Figure 2B–E) and *in vivo* (Figure 3). This triple reporter construct was successfully used to quantify the therapeutic responses of MDA-MB-231 human breast cancer tumors to the chemotherapeutic agents MMAE and MMAF by fluorescence and bioluminescence optical imaging (Figure 4A and B). The final *in vivo* optical signals of tumors directly correlated with the *ex vivo* tumor weights for all therapy groups (Table 2, Figure 5). These results validate our triple reporter as an accurate and valuable tool for imaging and quantifying *in vivo* tumor growth and response to therapy. This is the first reported use of both fluorescence and bioluminescence signals from a multi-reporter construct to measure drug efficacy *in vivo*.

Currently, the standard method for measuring cancer growth and regression *in vivo* is the use of calipers. One issue with caliper measurements is that these measurements are typically used to generalize the tumor volume from measurements in only two dimensions with the equation [0.5× (largest diameter) × (smallest diameter)^2^] [18], [19]. While tumors can sometimes be measured with calipers in three dimensions for a more accurate estimate of the tumor size [20], the orthotopic tumors in our study were often too small, especially in the MMAE-treated group, to obtain an accurate, consistent measurement of their heights through the mammary fat pads. Additionally, while there was a strong correlation between the caliper measurements and the fluorescence and bioluminescence signals, indicating that the fluorescence and bioluminescence signals are at least as good as the standard methods for measuring tumors, caliper measurements could not be attempted for tumors in the MMAE-treated group after day 19. Even though calipers could not be used to detect additional tumor shrinkage after day 19 in that treatment group, the tumors were consistently detectable by fluorescence and bioluminescence. Thus, a major advantage of the triple reporter system is that we can continue tracking and monitoring tumors when they are too small to be assessed with calipers. Also, calipers cannot be used to measure internal tumors *in vivo*, such as orthotopic pancreatic and bladder tumors, making reporter constructs essential for evaluating those models [21], [22]. Furthermore, caliper measurements can be inconsistent or systematically biased [23] because calipers are manually tightened and loosened around the tumor to determine size, which is operator-dependent. Finally, accurate caliper measurements require reliably palpable tumors; thus, the first treatment evaluation often does not start until the tumors are ∼100 mm^3^ [19], [24], [25].

By using optical signals from our triple modality reporter, we were able to sensitively track tumor growth and response to therapy by fluorescence and bioluminescence from the pre-palpable stage to large tumors up to 551 mm^3^ based on caliper measurements. Optical signals allowed for unbiased quantification of tumor therapy responses and for accurate tumor measurements in the MMAE-treated group throughout the full therapy experiment (Figure 4C). Additionally, the use of the triple reporter to monitor treatment allows for earlier detection of treatment responses when tumors have proportions more similar to those in human patients.

In summary, the engineering of cell lines expressing all three modalities of our optimized triple reporter allowed for sensitive, long-term quantification of tumor growth *in vivo* by fluorescence, bioluminescence, and PET imaging. This triple reporter construct has been optimized from previous designs [2]–[5] with the far-red fluorescent protein E2-Crimson for fluorescent imaging in the far-red part of the visible light spectrum; codon-optimized luciferase Luc2 for bioluminescence with improved sensitivity; and truncated wild type herpes simplex virus I thymidine kinase for PET imaging. Of note is that although E2-Crimson proved the best of the four candidate fluorescent proteins, its tetrameric structure means that it is not ideal for fluorescence resonance energy transfer or fusion to functional proteins. Additionally, short self-cleaving viral 2A sequences separate each component of the three reporter construct without requiring protein fusion or IRES sequences [11]. With these spacers, detection limits for red fluorescence required approximately 5-fold more cells than for bioluminescence (Figure S1), but fluorescence correlated more tightly than bioluminescence with tumor weight (Figure 5). The results of the MMAE and MMAF therapy experiment validate the use of the optimized triple modality reporter as an accurate and quantifiable *in vivo* imaging tool. Applications of this triple reporter to other tumor models could be useful for quantifying internal tumor growth or tracking tumor metastasis.

## Methods

### Construction of the optimized triple modality reporter and other control constructs

Individual cDNA encoding E2-Crimson (Clontech, Mountain View, CA), Luc2 (Promega), and wttk (gift from Professor Sanjiv Gambhir, Stanford University) were amplified using multiple overlapping primers with graded concentrations, to incorporate unique restriction sites, flexible Gly-Ser-Gly-linkers (GGGSGGG), and 2A sequences (P2A: ATNFSLLKQAGDVEENPGP and T2A: EGRGSLLTCGDVEENPGP) at the 5′ and 3′ ends in a single round of PCR. Subsequent gene stitching via overlap extension PCR yielded the following bicistronic cassettes adjoined by a Gly-Ser-Gly-linker-2A sequence: *NheI*-E2-Crimson-*BsiWI*-GSG-P2A-*HindIII*-Luc2-stop-*BamHI* and *HindIII*-Luc2-*BamHI-*GSG-T2A-*XhoI-*wttk-stop-*XbaI.* Amplified products were sequence verified before insertion into the pcDNA3.1/Hygro(+) vector (Life Technologies, Grand Island, NY) using *NheI/BamHI* or *HindIII/XbaI*, respectively. Finally, the Luc2-T2A-wttk-stop fragment was subcloned into pcDNA3.1/Hygro(+)_E2-Crimson-P2A-Luc2-stop plasmid using *HindIII* and *XbaI* restriction sites to generate pcDNA3.1/Hygro(+)_E2-Crimson-P2A-Luc2-T2A-wttk. The integrity of the tricistronic cassette and flanking restriction sites were confirmed by sequence analysis in the final triple modality reporter construct.

To generate other candidate triple modality reporters and bicistronic expression control constructs, cDNA encoding IFP, mNeptune (gift from Dr. Michael Z. Lin, Stanford University), and mPlum were amplified with *NheI* and *BsiWI* at the 5′ and 3′ ends, respectively, and inserted in place of E2-Crimson in the appropriate vectors via subcloning. E2-Crimson, IFP, mNeptune, mPlum, Luc2, and wttk, each encoding a stop codon, were subcloned into pcDNA3.1/Hygro(+) individually to make monocistronic control vectors.

### Comparison of the far-red and infrared fluorescent proteins

E2-Crimson, IFP, mNeptune, and mPlum, all inserted into the pcDNA3.1/Hygro(+) backbone, were transfected into HT-1080 cells (American Type Culture Collection, ATCC) with Lipofectamine2000 (Life Technologies) and selected for stable fluorescent protein expression with 3 weeks of hygromycin B (Sigma) treatment (from 50–500 µg/ml, dose increased every 3 days). A FACS Vantage SE Diva (BD Biosciences) was used to assess the population fluorescence with a 568 nm laser and 660/20 nm emission filter (E2-Crimson, mNeptune, and mPlum) and a 690 nm laser and 710–900 nm emission filter (IFP) at 100 mW power. The 5% brightest cells (1×10^6^) were subcutaneously implanted into athymic nude mice (female, 6-weeks-old), and the tumors were imaged 3 days later at ex 590/23 nm and em 645LP (E2-Crimson, mNeptune, and mPlum) or ex 640/48 nm and em700 LP (IFP) in a Maestro imager (CRi).

This study was carried out in accordance with the Guide for the Care and Use of Laboratory Animals of the National Institutes of Health recommendations. The UCSD Institutional Animal Care and Use Committee approved of all animal studies (Protocol: S04011). All imaging and injections were performed under isoflurane anesthesia.

### Comparison of triple reporter fluorescence and bioluminescence sensitivity

The triple reporter cDNA was transfected into HT-1080 cells (ATCC) with Lipofectamine2000 and selected for stable fluorescent protein expression as described in the previous section. A FACS Vantage SE Diva was used to collect the 5% brightest cells (100 mW laser with ex 568 nm and em 660/20 nm).

Athymic nude mice (female, 6-weeks-old) were implanted with subcutaneous HT-1080 triple reporter tumors (500–5000 cells/tumor in matrigel). Whole body fluorescence and bioluminescence was imaged using an IVIS Spectrum (Caliper Life Sciences) starting 5 minutes after tumor cell implantation. Mice were anesthetized with 2.5% isoflurane in 100% oxygen carrier gas. Fluorescence background signal was imaged with a 465 nm excitation filter and a 660 nm emission filter (FOV: D; binning: medium; f stop: 2; and exposure time: 1 second). E2-Crimson fluorescence signal was imaged with a 605 nm excitation filter and a 660 nm emission filter (FOV: D; binning: medium; f stop: 2; and exposure time: auto). Fluorescence background signal was subtracted from E2-Crimson fluorescence signal. Bioluminescence background was imaged with the excitation filter blocked and emission filter open (FOV: D; binning: medium; f stop: 1; and exposure time: 1 second). Then, mice were injected subcutaneously on the flank with 150 mg of D-luciferin in PBS (phosphate buffered saline) per kg of mouse. Mice were imaged 15 minutes after injection with the excitation filter blocked and emission filter open (FOV: D; binning: medium; f stop: 1; and exposure time: auto).

### Lentiviral cloning and production

The lentiviral vector plasmid, packaging psPAX2 plasmid, and pMDG.2 plasmid for VSV-G (vesicular stomatitis virus G) glycoprotein expression were generous gifts from Professor Didier Trono (Ecole Polytechnique Fédérale de Lausanne). The triple reporter cDNA was subcloned into the lentiviral vector plasmid with PCR amplification and standard cloning techniques. The transgene was inserted between a CMV promoter and WPRE (woodchuck hepatitis virus posttranscriptional regulatory element) sequence.

The HEK293A cell line (Life Technologies) was grown to 85% confluence in 15 cm culture dishes in high glucose DMEM media (Corning) supplemented with 10% fetal bovine serum and 1% penicillin/streptomycin (Corning) at 37°C in a 5% CO_2_ incubator. Before HEK293A transfection, the media in the culture was replaced with viral production media (high glucose DMEM media, 5 mM sodium butyrate, and 1% penicillin/streptomycin). The triple reporter lentiviral plasmid (24 µg), psPAX2 plasmid (45 µg), and pMDG.2 plasmid (30 µg) were added to 6 ml of Opti-MEM media (Life Technologies). Another 6 ml of Opti-MEM media containing polyethylenimine (0.5 mM; Sigma-Aldrich) was added to the 6 ml of Opti-MEM media containing the plasmids, and the mixture was incubated at room temperature for 15 minutes. An aliquot of this final solution (2 ml) was added to each dish. After 10 hours, media from each dish was replaced with fresh viral production media. The virus-containing media was collected at 48 and 72 hours after transfection and pooled for subsequent purification and concentration steps.

The pooled virus-containing media was filtered through a 0.45 µm filter (Millipore). The media was placed on top of a sucrose layer (20% sucrose in PBS) in centrifuge tubes and centrifuged in a SW28 rotor (Beckman Coulter) at 25,000 rpm (82,700 g) and 4°C for 2 hours. Each lentivirus pellet was resuspended in 40 µl of PBS for immediate use or stored at −80°C.

### Lentiviral transduction and FACS of the triple reporter cancer cell lines

MDA-MB-231 and HT-1080 cancer cells (ATCC) were grown to 85% confluence in 25 cm^2^ tissue culture flasks in complete growth media (EMEM media [ATCC], 10% fetal bovine serum, and 1% penicillin/streptomycin) at 37°C in a 5% CO_2_ incubator. Media from each flask was replaced with 5 ml of high glucose DMEM media supplemented with 5 mM sodium butyrate, 0.1 mM PEI, and 40 µl of the triple reporter lentivirus. After 12 hours, the viral media was replaced with complete growth media, and the cell lines were expanded in culture for 3 weeks. A FACS Vantage SE Diva was used to collect the top 1.5% brightest cells from each cell line based on E2-Crimson fluorescence. The MDA-MB-231 and HT-1080 FACS populations were expanded in culture for 3 weeks before use in all subsequent experiments.

### *In vitro* E2-Crimson, Luc2, and wttk activity

Cells were imaged for E2-Crimson expression with an epifluorescence microscope (Zeiss) using ex 580/20 nm, em 653/95 nm, and a 40× oil objective with a 1 second exposure time.

To confirm Luc2 activity, 7.4×10^4^ cells were grown in each well of a 48-well tissue culture plate with complete growth media for 24 hours. Media was replaced with 100 µl of PBS, and D-luciferin (In Vivo Imaging Solutions) was added immediately before imaging at a final working concentration of 150 µg/ml. The plate was imaged with an IVIS Spectrum (FOV: C; binning: medium; f stop: 1; and exposure time: auto). Each cell line was tested in triplicate.

Wttk activity was confirmed by ganciclovir treatment. Cells (4×10^3^) were grown in each well of a 96-well tissue culture plate with complete growth media supplemented with either 0 µg/ml, 1 µg/ml, or 10 µg/ml ganciclovir (InvivoGen) for 6 days. Cells were observed with bright field microscopy to visually assess cell death by ganciclovir treatment. Cell death from ganciclovir treatment was then quantified using the CellTiter 96 AQueous One Solution Cell Proliferation Assay (Promega). Results of this colorimetric method for determining the live cell count were collected using an Infinite M1000 PRO plate reader (Tecan) measuring absorbance at 490 nm in the bottom read mode. The cell viability was then calculated by comparing the absorbance readings from the ganciclovir treatment with the untreated cells. Each cell line was tested in triplicate.

### Western blot analysis

Cells (1.5×10^3^) from each cell line were lysed in RIPA buffer (Cell Signaling Technology) with 0.5% SDS and complete protease inhibitors (Roche) by mechanical disruption and freeze thawing; the cell mixtures were then reduced and denatured in NuPage LDS Sample Buffer (Life Technologies) with 8% β-mercaptoethanol, at 95°C for 5 minutes. The samples were run next to the Precision Plus Duel Color Standards (Bio-Rad) in a 4–12% Bis-Tris polyacrylamide gel (Life Technologies). Expression of each reporter protein and self-cleavage of the viral 2A sequences were evaluated by Western blots with a DsRed rabbit polyclonal antibody (Clonetech) diluted 1∶3000, firefly luciferase mouse monoclonal antibody (Abcam) diluted 1∶3000, HSV-1 thymidine kinase goat polyclonal antibody (Santa Cruz Biotechnology) diluted 1∶250, and GAPDH rabbit polyclonal antibody (Sigma-Aldrich) diluted 1∶5000. Secondary antibodies used were a goat anti-rabbit IgG HRP conjugate diluted 1∶3000 (Cell Signaling Technologies), goat anti-mouse IgG HRP conjugate diluted 1∶3000 (Bio-Rad), and donkey anti-goat IgG HRP conjugate diluted 1∶2500 (Promega).

### *In vivo* optical imaging of the triple reporter

Athymic nude mice (female, 6-weeks-old) were implanted with bilateral tumors, orthotopically into the mammary fat pads with the MDA-MB-231 triple reporter cells (1×10^6^ cells/tumor in matrigel) or subcutaneously at the shoulder blades with the HT-1080 triple reporter cells (5×10^5^ cells/tumor in PBS). These locations were chosen to separate the non-specific ^18^F-FHBG signal detected within the intestinal tract in the abdomen. The tumors were grown for 2 weeks.

Whole body fluorescence and bioluminescence was imaged using an IVIS Spectrum, as described in the comparison of triple reporter fluorescence and bioluminescence sensitivity section. The total radiant efficiency (p/s)/(cm^2^/sr) and total radiance photons (p/s) were quantified by drawing ROIs over the tumor using Living Image software (Caliper Life Sciences).

### *In vivo* PET imaging of the triple reporter

The David Stout laboratory in the Crump Institute for Molecular Imaging at Univeristy of California, Los Angeles synthesized 9-(4-[^18^F]Fluoro-3-hydroxymethylbutyl)guanine (^18^F-FHBG) and directed all microPET experiments [26], [27].

Mice were warmed for 20 minutes prior to tail vein injection of ∼150 uCi of ^18^F-FHBG. After 2 hours for specific uptake and non-specific clearance, mice were anesthetized using 2% isoflurane in 100% oxygen carrier gas and heat supported throughout the entire imaging process. Mice were imaged using a multimodality chamber designed to maintain anesthesia, heating, and reproducible positioning during all scans.

PET scans were acquired for 10 minutes using an Inveon DPET system (Siemens Preclinical Solutions). Images were reconstructed using filtered backprojection to a resolution of ∼1.8 mm. Images were reconstructed without attenuation or scatter correction because these effects are fairly small in mice and not necessary for this experiment. CT images were acquired immediately after PET scans using a MicroCAT II small animal CT system (Siemens Preclinical Solutions). The exposure settings were 70 kVp, 500 mAs, 500 ms exposure time, and 360° rotation in 1° steps with 2.0 mm aluminum filtration. Images were reconstructed using a modified Feldkamp process to a cubic voxel size of 0.20 mm. PET and CT images were automatically coregistered and stored in a single file.

Wttk activity was quantified by the% injected dose of ^18^F-FHBG uptake in tumors. Volumetric ROIs were drawn around the tumors as well as the whole body regions of the mice using AMIDE software [28]. Volumetric ROIs measured ^18^F-FHBG uptake as well as the size in mm^3^. The% injected dose was calculated by dividing the tumor ^18^F-FHBG uptake by the whole body ^18^F-FHBG uptake. The% injected dose per mm^3^ of the wild type (untransduced) tumors was subtracted from the% injected dose per mm^3^ of the triple reporter tumors to remove the background PET-CT signal.

The UCLA Institutional Animal Care and Use Committee approved of all animal studies (Protocol: 2006–135).

### Quantifying the therapeutic response of triple reporter tumors *in vivo* and *ex vivo*

Athymic nude mice (female, 5-weeks-old) were implanted with bilateral MDA-MB-231 triple reporter tumors (7.5×10^5^ cells/tumor in matrigel) orthotopically in the mammary fat pads. After 7 days, mice were randomized into 3 treatment groups (untreated, MMAE-treated, and MMAF-treated) with 5 mice per group for a total of 10 tumors per group. Mice were administered 0.5 nmol drug per gram mouse every 3 days for 6 doses on days 7, 10, 13, 16, 19, and 22. Whole body fluorescence and bioluminescence images were captured with an IVIS Spectrum and quantified using Living Image software, as described earlier. The total fluorescence and bioluminescence signals of the tumors were averaged within each group. The tumor size was also measured using millimeter calipers (the largest diameter and smallest diameter were measured; the tumor size was estimated as 0.5× [largest diameter] × [smallest diameter]^2^) starting on day 16, which is when the tumors in the untreated and MMAF-treated groups were reliably palpable. The mouse weight was recorded throughout the experiment. The tumors were resected and weighed 4 weeks post implantation. The tumor weights were averaged for each group for comparison with the final *in vivo* average fluorescence and bioluminescence signals.

### Statistical analyses

Statistical analyses were conducted with a 2-way ANOVA (analysis of variance) with multiple comparisons after the data were log-transformed. All results are given as the mean ± standard deviation. P<0.005 was considered statistically significant.

### Ethics statement

This study was carried out in strict accordance with the recommendations in the Guide for the Care and Use of Laboratory Animals of the National Institutes of Health. The protocol was approved by the Committee on the Ethics of Animal Experiments of the University of California San Diego (Protocol Number: S04011) and the Committee on the Ethics of Animal Experiments of the University of California Los Angeles (Protocol Number: 2006-135). All imaging and injections were performed under isofluorane anesthesia, and all efforts were made to minimize suffering.

### Accession code

The triple modality reporter GenBank accession number is KJ561464.

## Supporting Information

Figure S1 **Comparison of fluorescence and bioluminescence sensitivity.** (A) Fluorescence and bioluminescence imaging of varying numbers of cells from HT-1080 cells expressing the triple reporter; bioluminescence could detect 500 cells while fluorescence required 2,500 cells for detection. (B) Confirmation of the bioluminescence sensitivity for as few as 500 cells in another athymic nude mouse with HT-1080 triple reporter cells. (a1–a6) represent 5,000 cells; (b1–3) are 2,500 cells; (c1–c3) are 1,000 cells; and (d1–d3) are 500 cells injected subcutaneously. Gut autofluorescence from the alphalpha chow is indicated by *. The fluorescence signal is shown as the radiant efficiency (p/s/cm^2^/str)/(mW/cm^2^). The bioluminescence signal is shown as the radiance photons (p/s/cm^2^/sr).
(TIF)

Table S1 **Sizes and optical signals for the HT-1080 triple reporter tumors not detectable by PET.** Five smaller HT-1080 tumors were not detectable by PET, but they still produced reliable fluorescence and bioluminescence signals.
(TIF)
