# Supplementary figures and images for: An Optimized Triple Modality Reporter for Quantitative In Vivo Tumor Imaging and Therapy Evaluation
Source: PLoS One. 2014 May 9;9(5):e97415. doi: 10.1371/journal.pone.0097415 (PMC4016317; doi:10.1371/journal.pone.0097415)

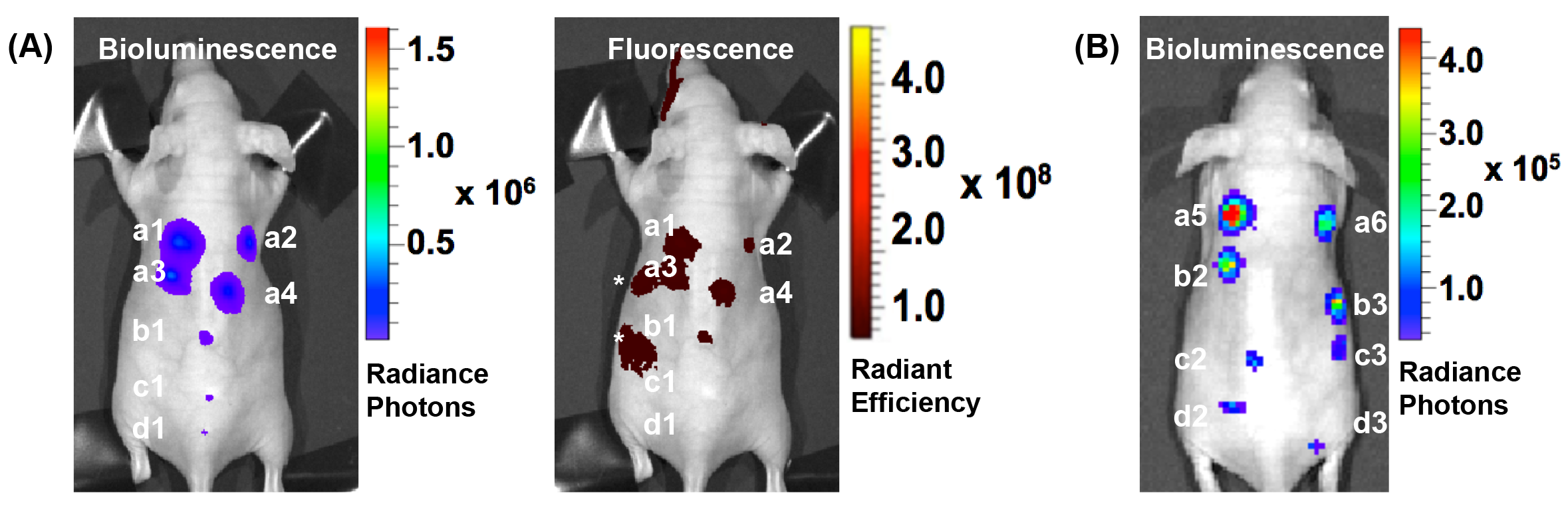

Supplement: Figure S1 — Comparison of fluorescence and bioluminescence sensitivity. (A) Fluorescence and bioluminescence imaging of varying numbers of cells from HT-1080 cells expressing the triple reporter; bioluminescence could detect 500 cells while fluorescence required 2,500 cells for detection. (B) Confirmation of the bioluminescence sensitivity for as few as 500 cells in another athymic nude mouse with HT-1080 triple reporter cells. (a1–a6) represent 5,000 cells; (b1–3) are 2,500 cells; (c1–c3) are 1,000 cells; and (d1–d3) are 500 cells injected subcutaneously. Gut autofluorescence from the alphalpha chow is indicated by *. The fluorescence signal is shown as the radiant efficiency (p/s/cm2/str)/(mW/cm2). The bioluminescence signal is shown as the radiance photons (p/s/cm2/sr). (TIF) [file pone.0097415.s001.tif]

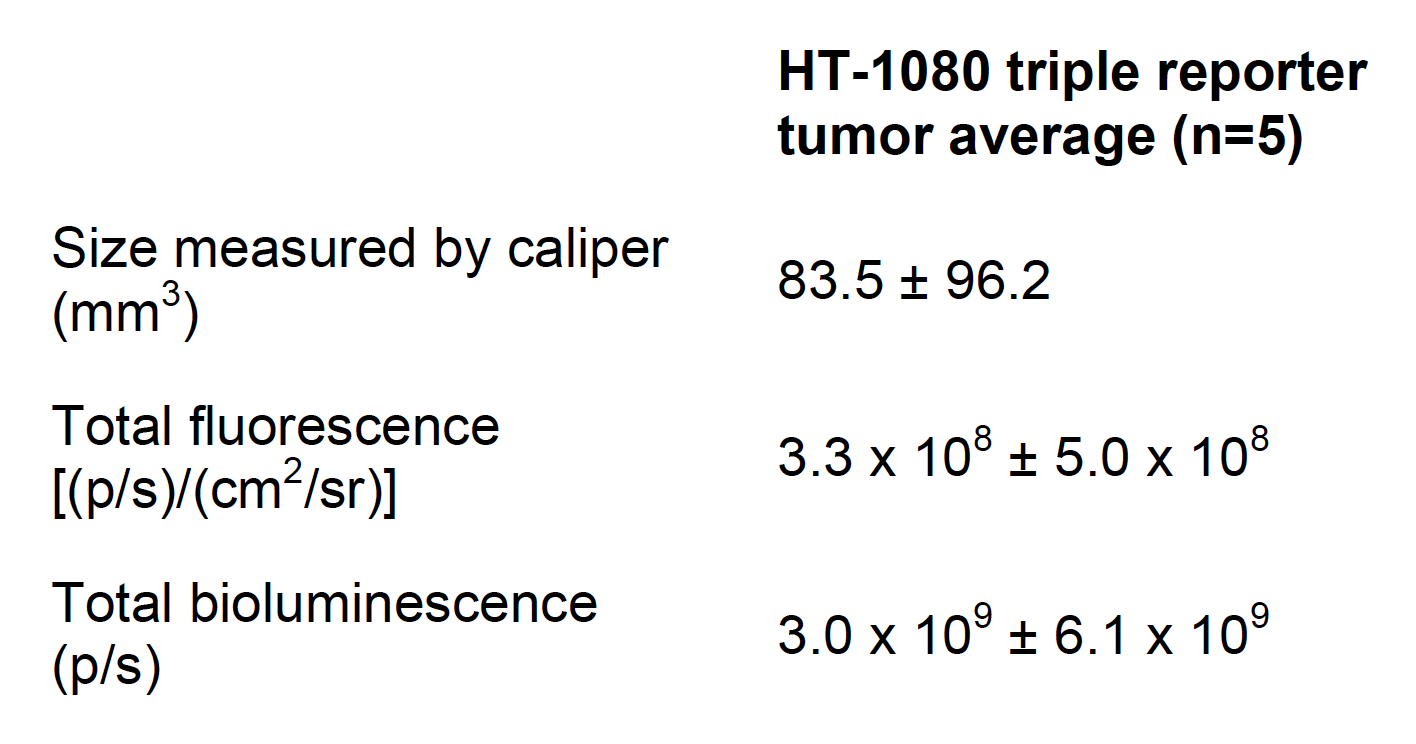

Supplement: Table S1 — Sizes and optical signals for the HT-1080 triple reporter tumors not detectable by PET. Five smaller HT-1080 tumors were not detectable by PET, but they still produced reliable fluorescence and bioluminescence signals. (TIF) [file pone.0097415.s002.tif]
